# Mammographic Breast Density in Pakistani Women, Factors Affecting It, and Inter-Observer Variability in Assessment

**DOI:** 10.7759/cureus.14050

**Published:** 2021-03-23

**Authors:** Kulsoom Fatima, Farwa Mohsin, Muhammad O Rao, Muhammad Ismail Alvi

**Affiliations:** 1 Radiology, Aga Khan University Hospital, Karachi, PAK

**Keywords:** breast density, mammogram, bi-rads, inter-observer variability, risk factors

## Abstract

Introduction

Breast density on mammography can affect the sensitivity of breast cancer detection and is an independent risk factor for breast cancer. The incidence of breast cancer in Pakistani women is reported to be the highest among women in Asia. No published data is describing the patterns of mammographic breast density in this population. We undertook this study to assess the Breast Imaging Reporting and Data System (BI-RADS) patterns of breast density on mammography, factors that affect breast density, and inter-observer variability in breast density assessment.

Methods

Bilateral breast mammograms were retrospectively reviewed for breast density by two separate readers (resident and attending radiologist). Breast density was categorized into four types according to the BI-RADS lexicon. Types 1 and 2 were grouped into non-dense and types 3 and 4 into dense breasts. The association of patient factors with breast density was assessed, with p < 0.05 considered statistically significant. The inter-observer variability in breast density assessment between the two readers was calculated using Cohen's κ coefficient.

Results

A total of 612 women underwent mammography in the study period. Type 3 (heterogeneously dense breast parenchyma) was the most frequent pattern (51.6%) followed by type 2 (scattered fibroglandular) pattern (38.9%). Fatty parenchyma (type 1) and extremely dense parenchyma (type 4) were the least common. Breast density was inversely related to age (p < 0.001) and parity (p <0.002). Breast density was also lower in postmenopausal women (p < 0.001). There was no statistically significant difference in mean age at menarche, age at first delivery, family history of breast cancer, or presence of cancer among women with dense and non-dense breasts. The inter-observer agreement was almost perfect (κ = 0.86).

Conclusion

The majority of women in our population (56.9%) had dense breasts (BI-RADS type 3 and 4) which decrease the sensitivity of breast cancer detection on mammography suggesting it may be insufficient as the sole screening/diagnostic tool in this population. Lower breast density was associated with increasing age, parity, and post-menopausal status. Breast density assessment was almost perfect among the resident and attending radiologist.

## Introduction

Mammography is the most sensitive tool in the detection of early breast cancer. The assessment of breast density is an important component of screening mammography. Breast density describes the proportion of dense to lucent areas on the mammogram indicating the amount of glandular tissue in the breast [1]. The sensitivity of mammography is affected by the amount of glandular tissue. Increased breast density may not only obscure small masses but is also considered an independent risk factor for breast cancer development [2-4].

It has been reported that the proportion of fatty tissue is higher in western women aged 40 years or more [5], making screening mammography a reasonable choice for early detection of breast cancer as recommended by the American Cancer Society [6]. Although the incidence of breast cancer is lower in Asia compared to western countries, Pakistan has the highest rate of breast cancer among Asian women, second only to Jewish women in Israel, with approximately 90,000 cases being diagnosed every year with a mortality rate of 44.44% [7]. There is however no data available regarding the patterns of breast density in Pakistani women and the factors affecting it, the knowledge of which is crucial as efforts are underway to develop a national screening program. Increased breast density also often leads to additional imaging. Breast density assessment performed according to the American College of Radiology Breast Imaging Reporting and Data System (BI-RADS) aims to reduce discordance in the interpretation of mammographic findings, standardize mammographic reporting, and facilitate follow-up [8]. However, this is a qualitative assessment and can be potentially subjective. Our study, therefore, also aimed to determine the reproducibility of breast density assessment between the resident and the attending breast radiologist. 

This article was previously presented as an abstract and poster at the 36th Annual Conference of Radiological Society of Pakistan (Virtual Meeting) held online from November 21-22, 2020 in Lahore, Pakistan.

## Materials and methods

**Figure 1:**
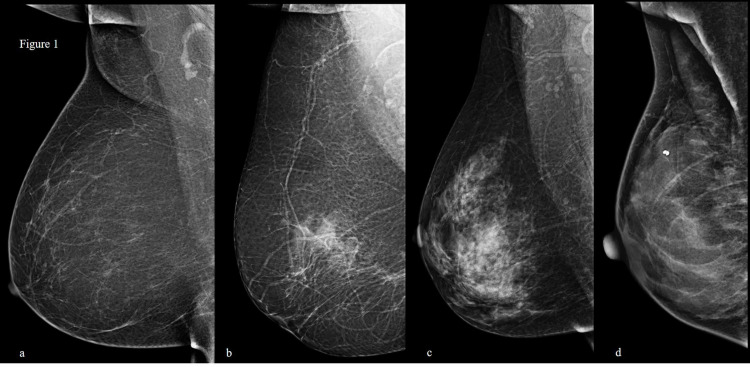
BI-RADS breast density patterns. (a) Type 1 - the breasts are "almost entirely fatty." (b) Type 2 - "there are scattered areas of fibroglandular density." (c) Type 3 - the breasts are "heterogeneously dense, which may obscure small masses." (d) Type 4 - the breasts are "extremely dense, which lowers the sensitivity of mammography." BI-RADS: Breast Imaging Reporting and Data System

After approval from the institutional ethical review committee, women undergoing bilateral mammograms from October 1, 2019, to December 31, 2019, at our hospital were identified. All mammograms were performed on Siemens Mammomat with PRIME technology (Siemens Healthineers, Erlangen, Germany). The parenchymal density on mammograms was assessed visually on both craniocaudal and mediolateral oblique views. If there was a difference in the density of both breasts, the higher density was recorded for the study. The breast density on mammograms was categorized into four types according to the BI-RADS® Atlas Fifth Edition (Figure 1) [8]. Mammograms with types 1 and 2 parenchymas were defined as non-dense while types 3 and 4 were defined as dense. 

All mammograms were reviewed independently by two readers (a third-year radiology resident and a credentialed attending radiologist with 12 years of experience in mammographic interpretation) to determine the mammographic breast density pattern. The readers were blinded to the indication of mammography and clinical factors. Inter-observer agreement between the two readers was calculated using Cohen’s κ statistic. The clinical data from a questionnaire completed at the time of mammography was recorded by a separate observer and included patient’s age, indication for mammography (screening or diagnostic), presenting complaint (if diagnostic), age of menarche, age at first delivery, parity, history of breastfeeding, use of hormone replacement therapy, family history of breast cancer, personal history of breast or any other cancer, and menopausal status.

All statistical analyses were performed using Statistical Package for the Social Sciences (SPSS) version 22.0 software (IBM, Armonk, NY). The independent t-test was used to compare continuous variables like mean patient age, mean age at menarche, and the mean age at first delivery between the women with dense and non-dense breasts. Pearson's chi-squared and Fisher’s exact test were used to compare the effect of selected risk factors among patients with dense and non-dense breasts. Two-tailed p-values were calculated. Results with p < 0.05 were considered statistically significant.

## Results

A total of 612 women underwent mammography in the study period; 462 (75.5%) were screening and 150 (24.5%) were diagnostic mammograms. There were 83 (13.6%) women younger than 40 years, 221 (36.1%) were 40-49 years old, 161 (26.3%) were 50-59 years old, 108 (17.6%) were 60-69 years old, and 39 (6.4%) were older than 70 years. Further, 59 (9.6%) women were nulliparous, 192 (31.4%) had one or two children, 271 (44.3%) had three or four children, and 90 (14.7%) had more than five children. 

The categorization of breast density by the two readers showed almost perfect agreement (κ = 0.86). All the disagreements were by one category only.

Breast density patterns

**Figure 2:**
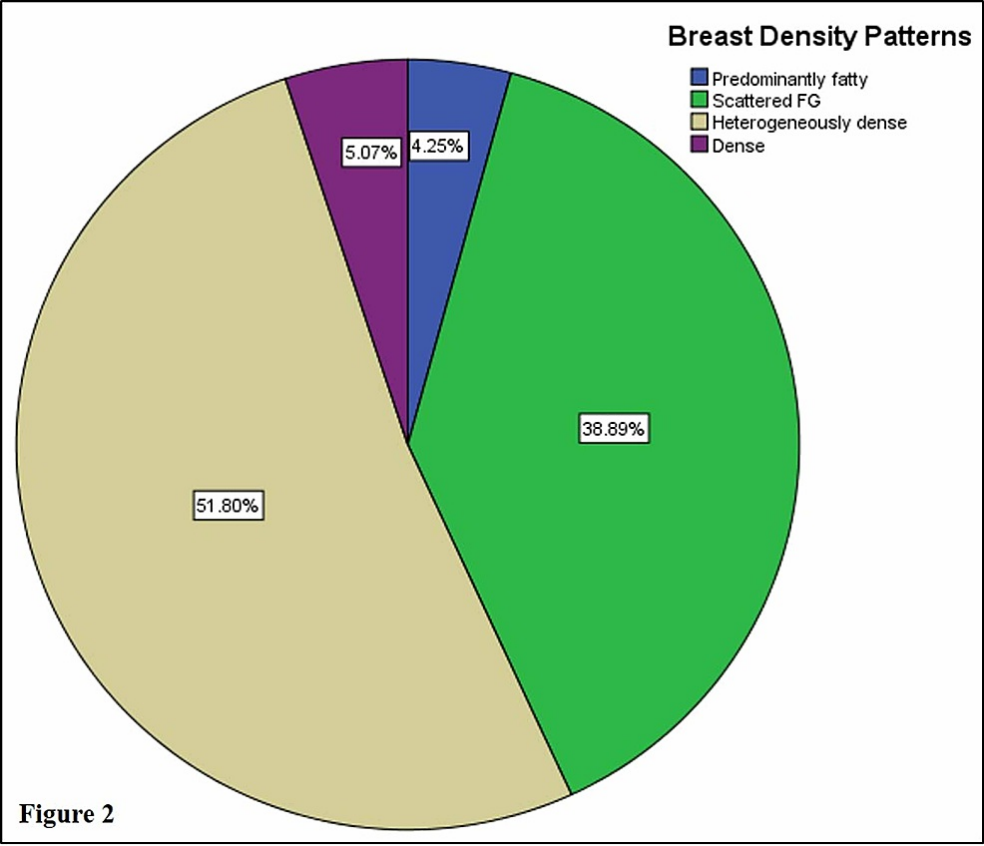
Percentage distribution of the four types of BI-RADS breast density patterns. FG: fibroglandular; BI-RADS: Breast Imaging Reporting and Data System

The most frequent parenchymal pattern seen was type 3 - heterogeneously dense breast parenchyma (51.80%). Figure 2 shows the distribution of the four breast density patterns. 56.9% of patients had dense breasts. 

Clinical factors associated with breast density

**Figure 3:**
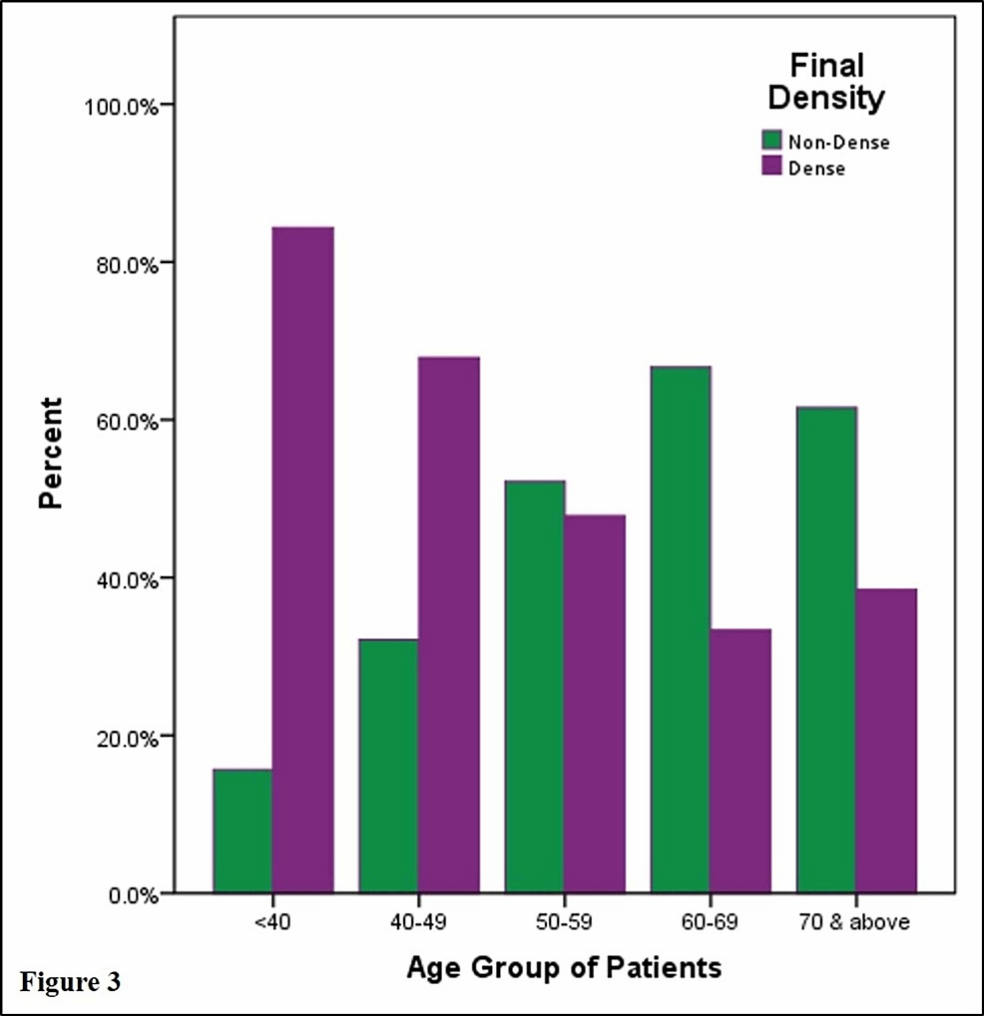
Percentage distribution of non-dense and dense breasts according to age groups.

**Figure 4:**
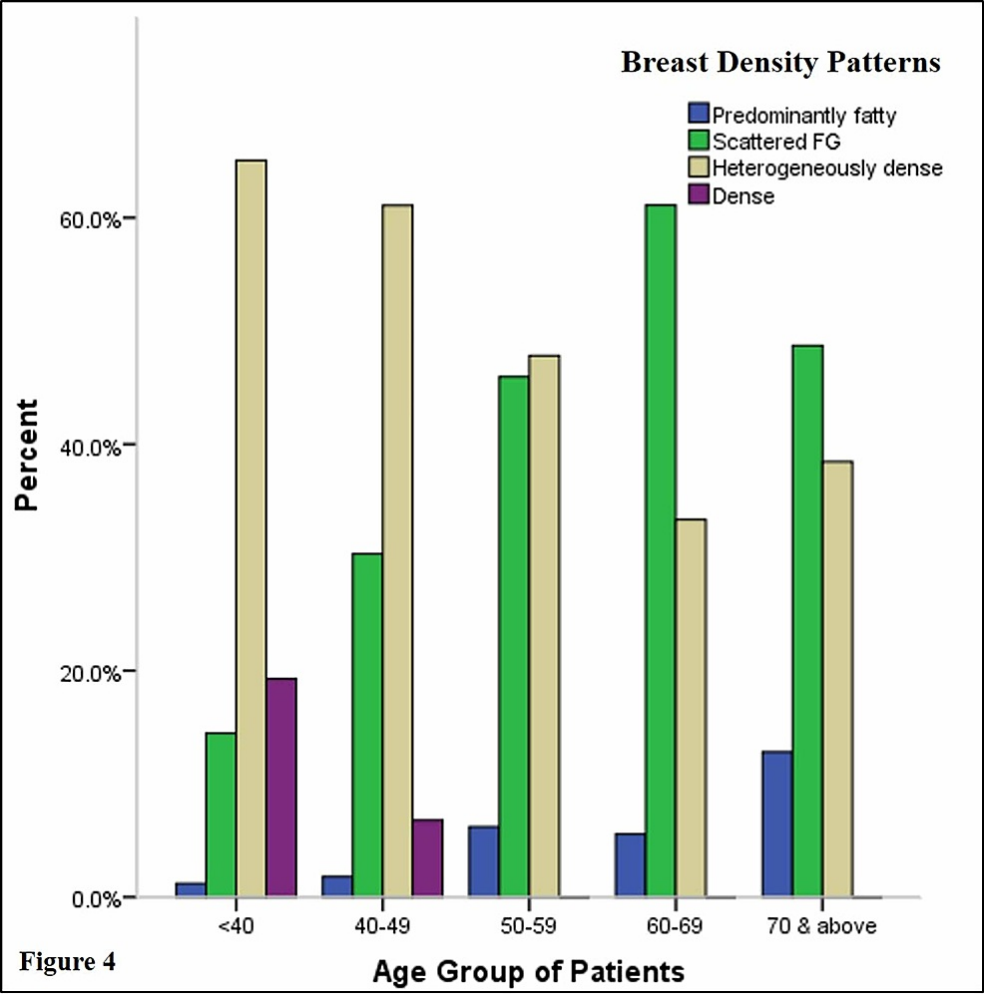
Distribution of BI-RADS breast density patterns according to age groups. FG: fibroglandular; BI-RADS: Breast Imaging Reporting and Data System

Breast density decreased with increasing patient age (Figure 3). The mean age of patients with dense breasts was 44.7 years while those with non-dense breasts was 55.4 years (p < 0.001). This variation in breast density was also statistically significant across the different age groups (Figure 4). Heterogeneously dense parenchyma predominated in the <40 years, 40-49 years, and the 50-59 years age cohorts while scattered fibroglandular parenchyma predominated in the 60-69 years and above 70 years age cohorts. Predominantly fatty parenchyma was the least common pattern in the <40 years age group (1/83, 1.2%) and was more prevalent with increasing age, with >70 years age group having the largest proportion (12.8%). Extremely dense parenchyma was only seen in <40 years (19.2%) and 40-49 years (6.7%) age cohorts. None of the women in the other age cohorts had extremely dense parenchyma. 

**Figure 5:**
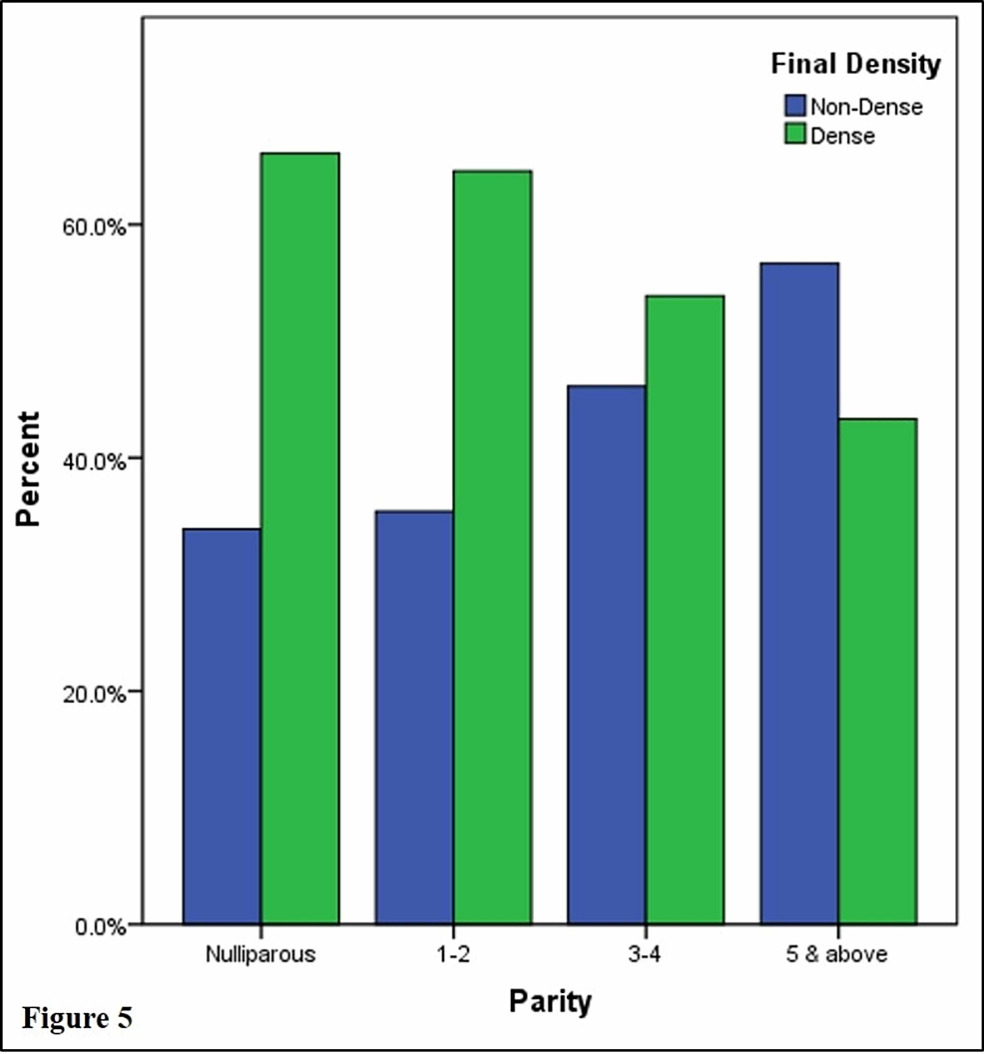
Percentage distribution of non-dense and dense breasts according to parity.

Breast density decreased with the increasing parity (p = 0.002; Figure 5). About two-thirds of nulliparous women (66.1%) and women with one to two children (64.5%) had dense breasts whereas most women with five or more children had non-dense breasts (56.6%).

The number of premenopausal women was 261 (42.6%), 333 (54.4%) were postmenopausal, and 18 women had a history of hysterectomy. Among the premenopausal women, 78.5% had dense breasts (p value < 0.001). There were no statistically significant differences in age at menarche, age at first delivery, and family or personal history of breast cancer between women with dense and non-dense breasts. Table 1 below compares clinical factors in women with dense and non-dense breasts.

**Table 1:** Breast density distribution according to selected patient factors.

Patient Characteristics	Breast Density		p-Value
	Non-dense (n = 264)	Dense (n = 348)	
Age group of patients (years)			<0.001
<40	13 (15.7%)	70 (84.3%)	
40-49	71 (32.1%)	150 (67.9%)	
50-59	84 (52.2%)	77 (47.8%)	
60-69	72 (66.7%)	36 (33.3%)	
>70	24 (61.5%)	15 (38.5%)	
Parity			0.002
Nulliparous	20 (33.9%)	39 (66.1%)	
1-2 children	68 (35.5%)	124 (64.5%)	
3-4 children	125 (53.5%)	146 (46.5%)	
5 or more children	51 (56.7%)	39 (43.3%)	
Menopausal status			<0.001
Pre	56 (21.5%)	205 (78.5%)	
Post	200 (60.1%)	133 (39.9%)	
Hysterectomy	8 (44.4%)	10 (55.6%)	
Personal history of breast carcinoma			0.91
Yes	45 (43.7%)	58 (56.3%)	
No	219 (43%)	290 (57%)	
Family history of breast carcinoma			0.21
Yes	90 (46.9%)	102 (53.1%)	
No	174 (41.4%)	246 (58.6%)	
Age at first delivery (years)			0.36
≤25	165 (45.6%)	197 (54.4%)	
>26	79 (41.4%)	112 (58.6%)	

## Discussion

Among the four patterns of breast density according to the BI-RADS lexicon, heterogeneously dense parenchyma, (type 3) was the most common pattern in our study and constituted more than 50% of the cases. Breast density on mammogram reflects the breast tissue composition. Fat appears as dark/lucent areas whereas fibroglandular tissue comprising acini and lobules along with the supporting connective tissue appears as light/white areas. Breast density has proven to be an independent risk factor for breast cancer by many investigators, and the reported risk is proportional to the degree of breast density [2,3,9]. Heterogeneously dense parenchyma, which is similar to type 4 parenchyma according to the Tabar classification, is reported to have more than twice the risk compared to other patterns [10,11], and the risk increases to four to five folds in women who have more than 75% dense parenchyma [2,3,12]. Besides being an independent risk factor, increased breast density also decreases the sensitivity of mammography as small masses may be obscured by the dense parenchyma [4,13]. Therefore, in such cases, mammography alone may be insufficient as a screening tool to detect breast cancer. Moreover, in a diagnostic setup, where a patient presents with a clinically suspicious lump, mammography may fail to detect satellite lesions in dense parenchyma, the presence of which changes the management significantly. Additional imaging, most commonly ultrasound, or magnetic resonance imaging (MRI) in selected cases, is employed to detect small masses in asymptomatic cases as well as to assess multifocality or multi-centricity in women with suspicious lumps and dense breasts. Ultrasound detects additional cancers when combined with mammography in women with dense breasts [14-16].

Several prior studies have demonstrated higher breast density in Asian women compared to non-Asian women [17-19]. Despite a higher proportion of dense breasts, the breast cancer incidence in Asia is lower than that in the United States or Europe [20,21]. This indicates the role of several other risk factors in breast cancer development besides breast density. Our study showed no statistical difference in breast density among women with a personal history of breast cancer and those without such history as shown in a study on the Chinese population [22]. Consistent with prior studies, we found a decrease in breast density with increasing age [1,22,23]. Also, breast density was higher in pre-menopausal women as previously shown [23,24]. The postulated reasons are post-menopausal regression of lobules and atrophy of the ductal epithelium as well as the intervening connective tissue with fatty replacement. Most women experience menopause at around 50 years and in our study also the reversal of breast density occurred in the 50-59 year age group.

Our study demonstrated a decrease in density with an increase in parity similar to those reported by prior studies [1,5]. The effects of parity are related to repeated breast involution after cessation of each lactating period. This study failed to demonstrate an association of breast density with age at menarche, age at first delivery, or family history of breast cancer. The association of age at menarche with breast density has been controversial, with few studies showing positive association while some showing negative or no association [25].

Our study showed almost perfect agreement between the breast density assessments by the two readers. This finding has important implications in our clinical environment as the mammograms are initially reviewed by the resident who suggests supplemental imaging based on the mammographic density. As more than half of the women have dense breasts, which decreases the sensitivity of mammography, they may undergo further imaging, usually ultrasound, without delay. Further study is needed to determine what proportion of women with dense breasts actually undergo additional imaging and the positive biopsy rate of lesions subsequently detected. Although this study assessed breast density qualitatively rather than quantitatively, the high level of the agreement indicates good reproducibility. Prior studies have shown a range of inter-observer agreements on breast density assessment from fair to almost perfect agreement [26-28]. 

There are limitations to our study. This is a single tertiary center study in an urban setting and the study population may not entirely represent Pakistani women. Also, we did not assess the use of hormone replacement therapy and body mass index which may potentially affect parenchymal patterns and breast density. Another limitation is that the number of cases with breast cancer and those without breast cancer was not matched which may have resulted in selection bias while assessing breast density among these two groups. Also, we included both first- and second-degree relatives in the family history of breast cancer, which may have attenuated the effect of this variable. 

## Conclusions

In this study of women in Pakistan, more than half of the subjects had dense breasts on mammography. Because dense breasts decrease the sensitivity of breast cancer detection, mammography may not be sufficient as the only screening or diagnostic tool in this population. Therefore, additional imaging tests such as ultrasound or MRI may be required for further evaluation of such women and should be considered when designing and implementing a national screening program. Breast density decreased with increasing age and higher parity and in post-menopausal women. Breast density assessment was almost perfect between the resident and attending radiologist.
